# Improved glycemic outcomes in people with type 2 diabetes using smart blood glucose monitoring integrated with popular digital health therapeutics

**DOI:** 10.1038/s41598-025-93605-1

**Published:** 2025-03-14

**Authors:** Mike Grady, Elizabeth Holt, Hilary Cameron, Steven Edelman

**Affiliations:** 1 LifeScan Scotland Ltd, Beechwood Park North, Inverness, IV2 3ED UK; 2 LifeScan Global Corporation, 20 Valley Stream Parkway, Malvern, PA 19355 USA; 3 UC San Diego School of Medicine, San Diego, CA USA

**Keywords:** Digital therapeutic, Health app, Type 2 diabetes, Lifestyle, Metabolic syndrome, Blood glucose monitoring, Endocrinology, Health care

## Abstract

The increasing prevalence of metabolic syndrome and type 2 diabetes places a burden on healthcare systems, necessitating cost-effective, engaging and accessible interventions to address the underlying behavioral and lifestyle drivers. Our study evaluated combining Bluetooth connected OneTouch blood glucose meters (BGM) and the OneTouch Reveal mobile app with one of four digital therapeutic apps. Each group was independent, with people with type 2 diabetes (PwT2D) themselves choosing their therapeutic intervention, to better reflect real-world use. Our 3-month decentralized study screened 912 subjects, with 612 returning mail-in A1cs, providing 191 subjects (Noom = 68, Fitbit = 31, Cecelia Health = 47, Welldoc = 45) who met all inclusion criteria, including entry A1c 7.5 to 12.0%. The primary endpoint of A1c change showed improvement in the overall group by − 0.77% (95% CI − 0.98 to − 0.56, *n* = 141) after 3-months, Noom − 1.03% (CI − 1.4 to − 0.61, *n* = 49), Fitbit − 0.56% (CI − 1.0 to − 0.11, *n* = 24), Cecelia Health − 0.76% (CI − 1.2 to − 0.36, *n* = 36), Welldoc − 0.55% (CI − 0.94 to − 0.17, *n* = 32). In terms of secondary endpoints, more than half (56%) of these PwT2D lowered A1c by ≥ 0.5% and more than a third (36%) lowered A1c by ≥ 1.0%, with similar improvements across each of the four independent groups. Our real-world approach shows the potential for connected BGMs and widely accessible digital therapeutics to contribute to improvements in glycemic outcomes.

**Supplementary Information:**

The online version contains supplementary material available at 10.1038/s41598-025-93605-1.

## Introduction

The increasing prevalence of metabolic syndrome and type 2 diabetes (T2D) is, in part, fuelled by modifiable lifestyle factors, such as lack of physical activity and less healthy food choices. A recent analysis of US NHANES data found the prevalence of metabolic syndrome increased from 37.6% in 2011 to 41.8% in 2018, with the authors concluding that people need to make lifestyle modifications to lower their risks for diabetes and cardiovascular disease^1^. Given the magnitude of the effort required to reach the millions of people in need of behavioral and/or lifestyle support, widely available tools, such as digital health therapeutics and apps, can play a key role in improving health outcomes. Despite this need, study designs that truly mirror how people interact with technology in a real-world setting are sparse, especially studies that allow subjects (and not investigators) to choose the devices they want to experience. An example is real-world data from people using Noom (a weight loss app) demonstrating that 49% of people maintained at least 10% weight loss after 1 year^2^.

In addition to focusing on weight loss, minimal physical activity has been observed to confer a 20% risk reduction in all-cause mortality compared to being physically inactive^3^, and regular aerobic exercise can reduce A1c by up to 0.7%^4^. There is also clinical evidence for significant health benefits, including reductions in A1c, in people using wearable activity trackers, such as Fitbit^5–7^. New incretin-based therapies for weight loss and diabetes management, such as Wegovy, Ozempic and Mounjaro, have surged in popularity but it is noteworthy that such therapies are intended as being complementary to, not a substitute for, healthy lifestyle choices and behaviors^8,9^.

A recent narrative review on technology use in primary care stated that technology should be individualized based on a patient’s needs, desires, skill level, and access^10^. Building on these points, we intentionally designed the ECLIPSE study (**E**viden**c**e for hea**l**th **i**mprovement in **p**eople with diabetes u**s**ing OneTouch & partn**e**r solutions) to mirror how people naturally access and experience digital solutions, allowing us to investigate the clinical benefits of four popular digital therapeutics (Noom, Fitbit, Cecelia Health and Welldoc) in combination with a Bluetooth connected OneTouch BGM. This current report focuses on the clinical outcomes achieved by PwT2D over a 3-month period.

## Methods

### Study details and inclusion criteria

Subject recruitment and management were provided by the virtual Contract Research Organization, Evidation Health Inc (San Mateo, CA, US, https://evidation.com/). The Evidation service included a patient portal hosting online recruitment from a database of PwT2D who had given prior consent to be contacted about clinical studies, online screening, gaining ethics approval (from the Advarra IRB, (Institutional Review Board)), managing supplies, and monitoring subject completion of study procedures and surveys. The Advarra IRB approved our research, and we confirm that our study was performed in accordance with relevant guidelines/regulations, including the Declaration of Helsinki, and that informed consent was obtained from all study subjects. ECLIPSE is registered at https://clinicaltrials.gov/study/NCT05354297. To supplement recruitment, we partnered with TCOYD (Taking Control Of Your Diabetes, San Diego, CA, US, https://tcoyd.org/), who created bespoke social media and email recruitment materials containing links to enrollment web pages on the Evidation portal. We also leveraged recruitment support from the diaTribe Foundation to publicize ECLIPSE on their Facebook, LinkedIn, and Instagram platforms (www.diaTribe.org).

Subjects were 18 years or older and living in the US with a self-reported diagnosis of T2D, a current A1c value of ≥ 7.5 and ≤ 12.0%, a body mass index ≥ 27 (based on self-reported height and weight), and self-reported use of at least 1 oral and/or injectable diabetes medication. Those who were pregnant or reported trying to conceive were ineligible for the study. Subjects were required to speak, read, and understand English and be currently using a BGM. Subjects were excluded who were currently using a OneTouch Verio Reflect BGM or any continuous glucose monitoring (CGM) system, or who had used a CGM in the last 3 months. For Fitbit subjects only, we excluded people currently using Fitbit Premium, MyFitnessPal, MapMyRun, or Withings, or who had used these apps in the last 3 months at least once per week. Excluding prior use of fitness apps allowed us to ensure subjects experienced setting and tracking activity goals without the benefit of significant prior knowledge. Enrolling subjects naive to the study devices and apps minimized bias from previous device use that would have confounded the reality of how people onboard, set-up, learn and engage with connected monitoring and digital devices.

### Statistical analysis

Analyses were performed using Python, R and IBM SPSS Statistics 21. All statistical hypothesis tests were conducted at the study-wide or familywise α = 0.05 level of statistical significance. Likewise, all confidence intervals (CIs) reflect the study-wide or familywise 95% confidence level. To maximize the robustness of the glycemic metric analysis, subject inclusion was limited to those with at least five readings during baseline (the first 14 days) and five readings during the 14 days prior to the 3-month timepoint. A complete case analysis was performed, so only those subjects who submitted their 3-month A1c sample were considered admissible for data analysis.

### Digital health and wellness therapeutic choices

**Fig. 1:**
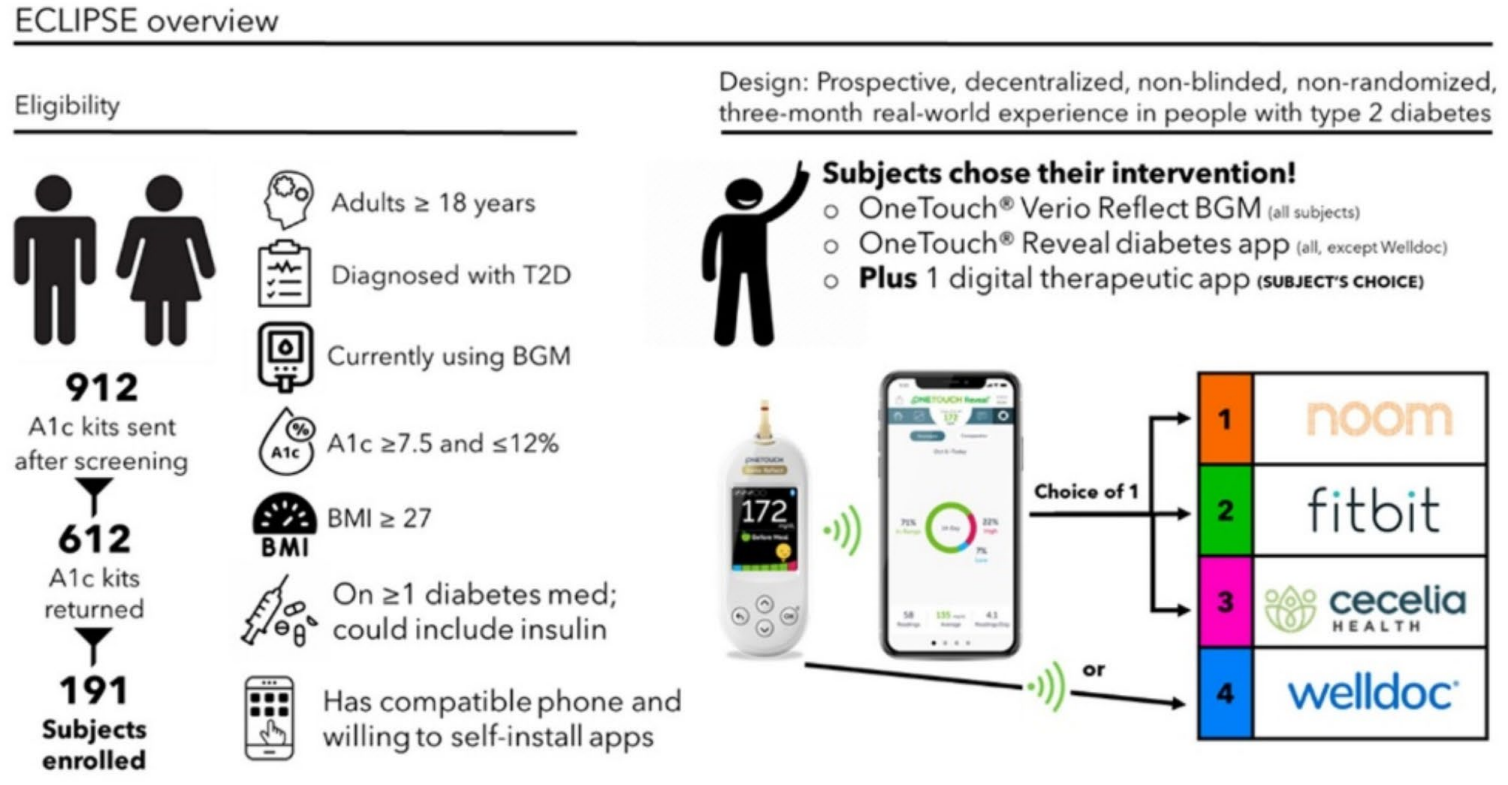
ECLIPSE overview.

Rather than employing a randomization scheme, subjects chose a preferred digital health and wellness therapeutic in combination with OneTouch diabetes management products (Fig. 1). There was no intention to compare outcomes between groups given that each digital therapeutic intervention was unique, and the motivations and needs of each subject were also unique. Subjects downloaded study digital therapeutic apps to their phone as instructed by Evidation Health.

All subjects received a Bluetooth connected OneTouch Verio Reflect (OTVR) blood glucose meter with a sufficient supply of test strips for 3 months in the mail and subjects in the Noom, Fitbit, and Cecelia Health groups downloaded the OneTouch Reveal (OTR) diabetes management app to their personal phone. The four digital health and wellness therapeutic intervention partners are described as follows (see Digital Supplement 1 for example screenshots from these digital therapeutic interventions):

- **Noom Diabetes Support** is a comprehensive behavior change program built upon the psychological principles of cognitive behavioral therapy. It is delivered via a smartphone-based app involving daily articles, logging of food, weight, exercise, and blood sugar, as well as interactions with a 1:1 coach and a support group. Subjects synced their OTVR meter to the OTR app. Subjects connected their OTR app to their Noom app to allow blood glucose readings to appear within the Noom app.
- **Fitbit** uses its Inspire 2 tracker and Fitbit Premium subscription to help encourage more movement, less stress, better eating habits and better sleep. Subjects received the Inspire 2 tracker and Fitbit premium app subscription. Subjects synced their OTVR meter to the OTR app and connected the OTR app to the Fitbit app to allow blood glucose readings to appear within the Fitbit app.
- **Cecelia Health** offers guidance from certified diabetes care and education specialists. Subjects synced their OTVR meter to the OTR app and used the OTR app to activate chat and allow Cecelia Health coaches to view blood glucose readings and aggregated glycemic metrics within the OTR app. Subjects could send texts to their Cecelia Health coach via the OTR app or choose live telephone coaching sessions with a Cecelia Health coach.
- **Welldoc** is a personalized health coaching and support program. Subjects synced their OTVR meter directly to the Welldoc app to allow their glucose readings to appear within the Welldoc app.

### Study procedures

Eligible subjects were sent a mail-in A1c test kit to be collected and returned to Molecular Testing Labs (https://moleculartestinglabs.com/) within 2 weeks of providing informed consent. Subjects completed an online survey to record demographics and medical history, and enrolled subjects were mailed sufficient diabetes supplies for 3 months. Subjects accessed online instructions on how to set up their new Bluetooth OTVR meter and sync it with their OTR or Welldoc app and gave permission for data transfer to the Sponsor. Subjects choosing the Fitbit group also received an Inspire 2 Fitbit tracker. Subjects were emailed relevant links and promotional codes to download and activate their chosen digital health and wellness therapeutic app or register for an online dashboard account with Cecelia Health.

### Data collection

The initial ECLIPSE design specified 6 months of data collection; however, due to slower than planned recruitment, the study was updated to a 3-month experience. Mail-in A1c tests were completed during enrollment (baseline) and after 3 months. Blood glucose readings from the OTR app were automatically sent to the LifeScan server for later analysis. Engagement data for each subject’s OTR app activity (number of sessions and time spent on the OTR app) were provided to us by Google Analytics. Each digital health partner sent weekly files of specified app activity and data from consented subjects directly to LifeScan to allow later determination of engagement with each digital therapeutic intervention. Weekly (and monthly) app interactions were tracked by each health partner using their own app software (Noom, Fitbit, Welldoc) or online patient/healthcare professional dashboard (Cecelia Health). We defined upfront that “active user” would mean performing such tasks as reading articles, logging meals, recording weight, tracking steps or interacting with a personal coach, all on a weekly basis. Subjects also responded to patient reported outcomes surveys via the Evidation online portal immediately after enrollment and at the 3-month mark, including: diabetes distress (T2DDAS-Core), a bespoke medication adherence survey, well-being (WHO-5), and patient activation (PAM-13).

## Results

### Overview of demography and medical history

**Fig. 2:**
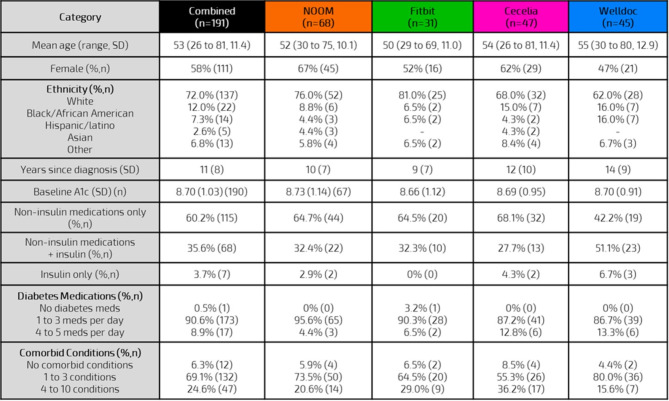
Baseline demographics.

The demographics and medical history of subjects were similar across the four independent groups. A total of 191 subjects fully enrolled in the study (Fig. 2): mean age 53 (SD 11.4) years, 58.0% (111/191) female, 72.0% (137/191) white, and living with T2D an average of 11 (SD 8.0) years. Non-insulin diabetes medications were taken by 60.2% (115/191), non-insulin diabetes medications and insulin were taken by 35.6% (68/191), and 3.7% (7/191) were on insulin therapy only. A majority (69.1%, 132/191) were living with 1 to 3 co-morbidities, with an additional 24.6% (47/191) having 4 to 10 co-morbidities (see Digital Supplement 2 for additional details on medications and co-morbidities). In terms of the overall cohort, 141 of the 191 subjects returned mail-in A1c kits at baseline and after 3 months. The primary endpoint, change in A1c, showed − 0.77% lowering after 3 months (95% CI − 0.98 to − 0.56) with 56% and 36% of these subjects achieving either a ≥ 0.5% or ≥ 1.0% reduction in A1c after 3 months.

### Primary and secondary endpoints per independent parallel group

#### OneTouch + Noom group

**Fig. 3:**
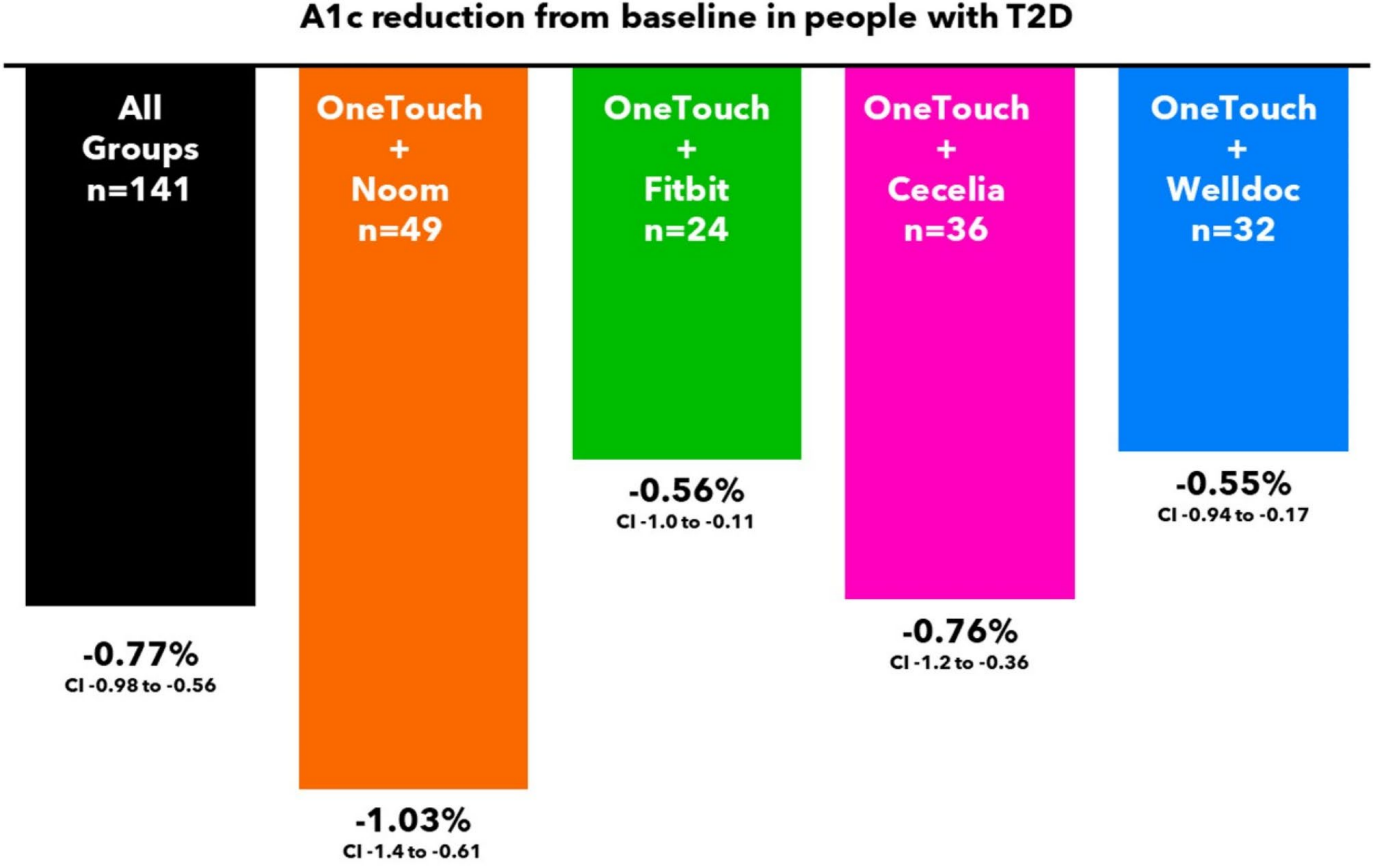
A1c reduction from baseline.

**Fig. 4:**
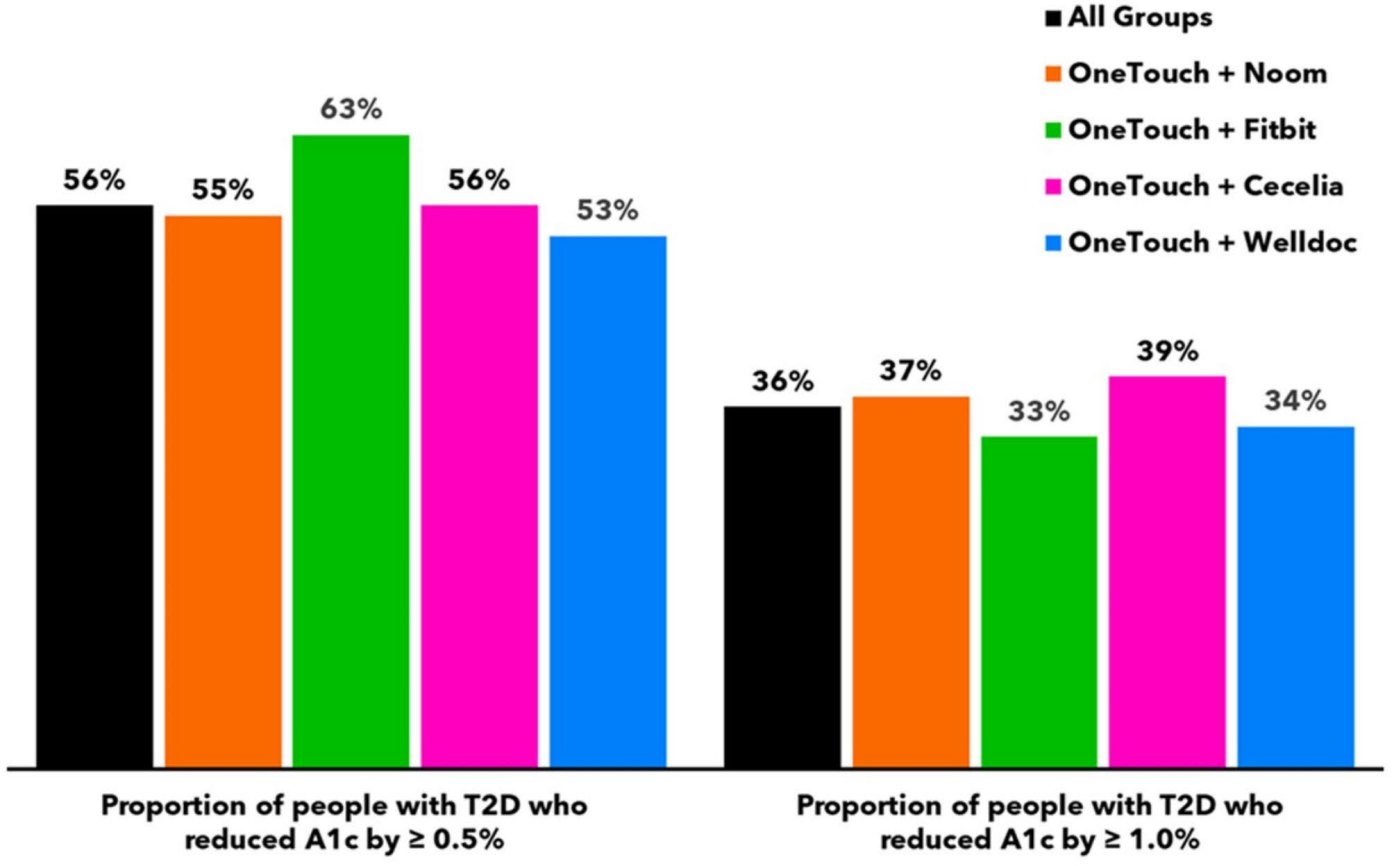
Proportions of people reducing A1c.

**Fig. 5:**
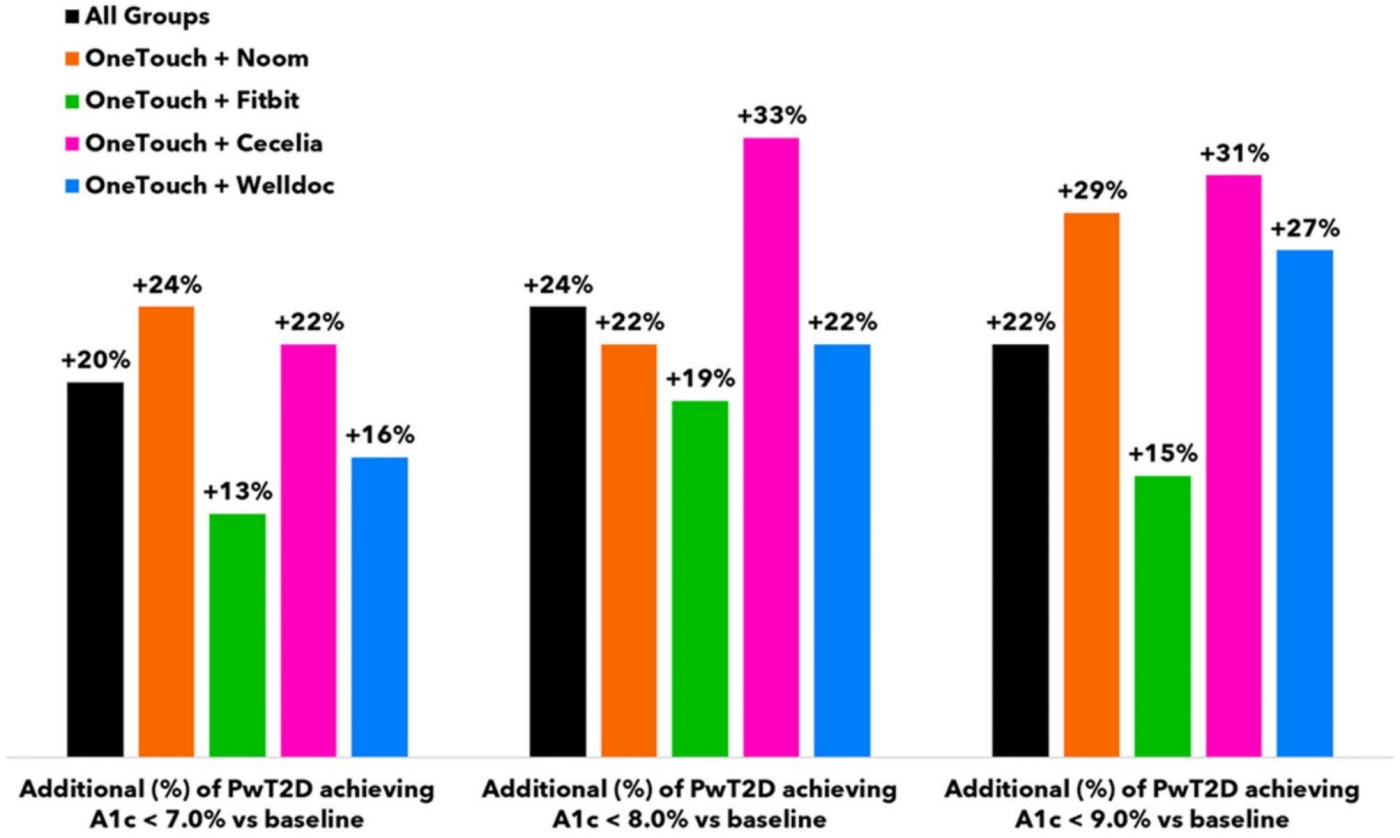
A1c target achievement.

**Fig. 6:**
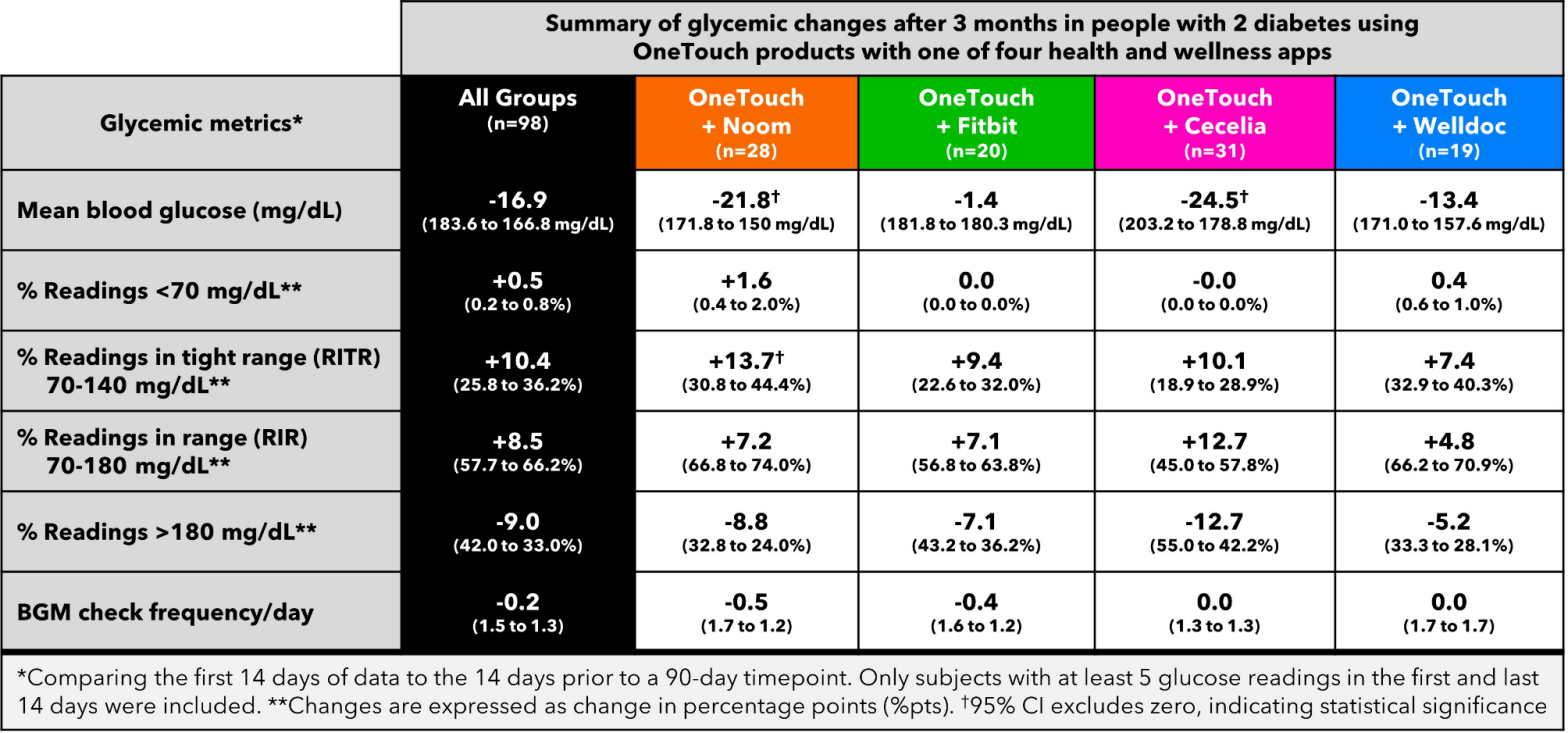
Summary of glycemic changes.

Subjects lowered A1c by − 1.03% from 8.73 to 7.69% (*n* = 49, 95% CI − 1.4 to − 0.61) after 3 months. Nineteen subjects (19/68) did not return their A1c test kit at 3 months, leaving 49 subjects for analysis of the primary and secondary endpoints. A1c improvements of ≥ 0.5% and ≥ 1.0% were seen in 55% (27/49) and 37% (18/49) of subjects, respectively. An additional 24%, 22% or 29% of subjects achieved HEDIS goals of an A1c < 7.0%, < 8.0 or < 9.0%, respectively, compared to baseline. For all 68 subjects, mean BG reduced by − 21.8 mg/dL (171.8 to 150.0 mg/dL, 95% CI, − 36.6 to − 7.0), readings in tight range (RITR) improved by + 13.7% points (%pts, 95% CI, 2.9 to 24.4) from 30.8 to 44.4% and readings in range (RIR) increased by + 7.2%pts from 66.8 to 74.0% but did not reach statistical significance (Figs. 3, 4, 5 and 6).

#### OneTouch + Fitbit group

Subjects lowered A1c by − 0.56% from 8.66 to 7.95% (*n* = 24, 95% CI − 1.0 to − 0.11) after 3 months. Seven subjects (7/31) did not return their A1c test kit at 3 months, leaving 24 subjects for analysis of the primary and secondary endpoints. A1c improvements of ≥ 0.5% and ≥ 1.0% were seen in 63% (15/24) and 33% (8/24) of subjects, respectively. An additional 13%, 19% or 15% of subjects achieved HEDIS goals of an A1c < 7.0%, < 8.0 or < 9.0%, respectively, compared to baseline. For all 31 subjects, mean BG was unchanged at − 1.4 mg/dL (181.8 to 180.3 mg/dL), RITR increased by + 9.4 %pts from 22.6 to 32.0% and RIR increased by + 7.1 %pts from 56.8 to 63.8%. Despite positive trends, changes in mean BG, RITR and RIR did not reach statistical significance (Figs. 3, 4, 5 and 6).

#### OneTouch + Cecelia health group

Subjects lowered A1c by − 0.76% from 8.69 to 7.82% (*n* = 36, 95% CI − 1.2 to − 0.36) after 3 months. Eleven subjects (11/47) did not return their A1c test kit at 3 months, leaving 36 subjects for analysis of the primary and secondary endpoints. A1c improvements of ≥ 0.5% and ≥ 1.0% were seen in 56% (20/36) and 39% (14/36) of subjects, respectively. An additional 22%, 33% or 31% of subjects achieved HEDIS goals of an A1c < 7.0%, < 8.0 or < 9.0%, respectively, compared to baseline. For all 47 subjects, mean BG reduced by − 24.5 mg/dL (203.2 to 178.8 mg/dL, 95% CI, − 46.8 to − 2.1), RITR increased by + 10.1%pts from 18.9 to 28.9% and RIR increased by + 12.7%pts from 45.0 to 57.8%. Changes in RITR and RIR did not reach statistical significance, although the trends were positive (Figs. 3, 4, 5 and 6).

#### OneTouch + Welldoc group

Subjects lowered A1c by − 0.55% from 8.70 to 7.98% (*n* = 32, 95% CI − 0.94 to − 0.17) after 3 months. Thirteen subjects (13/45) did not return their A1c test kit at 3 months, leaving 32 subjects for analysis of the primary and secondary endpoints. A1c improvements of ≥ 0.5% and ≥ 1.0% were seen in 53% (17/32) and 34% (11/32) of subjects, respectively. An additional 16%, 22% or 27% of subjects achieved HEDIS goals of an A1c < 7.0%, < 8.0 or < 9.0%, respectively, compared to baseline. For all 45 subjects, mean BG reduced by − 13.4 mg/dL (171.0 to 157.6 mg/dL), RITR increased by + 7.4%pts from 32.9 to 40.3% and RIR increased by + 4.8%pts from 66.2 to 70.9%. Changes in mean BG, RITR and RIR did not reach statistical significance (Figs. 3, 4, 5 and 6).

### Subject engagement with digital therapeutics

**Fig. 7:**
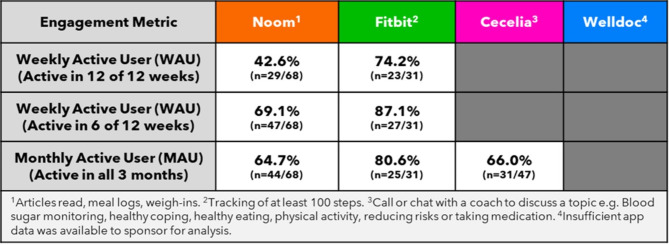
App engagement metrics.

Data from the digital partners provided insights on the in-app actions performed by subjects. In the Noom group, 69.1% (47/68) were active in the app at least once weekly, including reading articles, logging meals or weighing in. In the Fitbit group, the app recorded at least 100 steps (a proxy that the Fitbit device was used) on one or more days per week in 87.1% (27/31). For Cecelia Health subjects, 66.0% (31/47) engaged at least once per month with their coach to set goals and track their progress. These goals fell under the themes of blood sugar monitoring, healthy coping, healthy eating, physical activity, reducing risks, or taking medication. Cecelia Health subjects set 2.7 goals on average and met 1.8 of them, with 77% (36/47) achieving more than 50% of the initial goals set over the full 3 months. There was insufficient app data available to the sponsor for an analysis of Welldoc in-app engagement (Fig. 7).

## Discussion

Our study observed clinically meaningful A1c reductions (ranging from − 0.55 to − 1.03%) in PwT2D experiencing one of four popular digital health therapeutics over a 3-month period. Noom, Fitbit, Cecelia Health and Welldoc represent some of the most prominent and globally recognizable digital health therapeutics used by people with (or without) diabetes. Our data further substantiates the value of these interventions and suggests what could be achieved by similar PwT2D under real-world conditions.

Our cohort reflected the significant co-morbidities and polypharmacy common to people who have been managing type 2 diabetes for a decade or more. Coexisting conditions, such as obesity, depression, hypertension, and dyslipidemia, contributed additional metabolic risk to our demographic, necessitating a multi-faceted approach to the interventions we offered. For this reason, our four carefully selected digital health therapeutic interventions were broadly focused on providing insights that stimulate positive behavior change to lower metabolic risks and improve general health and well-being. However, our key priority and primary endpoint was improving diabetes management, and therefore, all subjects switched from their current BGM to a Bluetooth connected OTVR meter and the OTR diabetes management app (the OTR app was not mandatory in the Welldoc group). There is synergy between some features present on OTR and the other four health apps (e.g., recording BG readings, food intake, or tracking medications) but the OTR app provides unique features, including the Blood Sugar Mentor feature, ColorSure blood glucose range indicator, automatic high or low glucose pattern recognition, and the ability to view or share a 14-day glycemic summary report to help understand how behavior, lifestyle and medication adjustments (e.g., insulin dosing) impact daily glucose profiles^11^.

A key principle of ECLIPSE was personal choice. We felt it would be counterproductive to randomize subjects because we wanted the intervention selected to serve the lifestyle needs of each subject. Forced randomization, common to many clinical studies, would not mimic how people naturally choose a digital health therapeutic at home and more importantly, would not take into account personal motivations for behavior change or health needs. In keeping with this philosophy, we viewed each of our interventions as a self-contained experience and did not seek to compare outcomes between groups.

Encouragingly, across all four interventions, 53 to 63% of subjects lowered A1c by ≥ 0.5%, 33 to 39% of subjects reduced A1c by ≥ 1.0% and, compared to baseline, an additional 13 to 24% of subjects achieved the guideline goal of A1c < 7.0%^12^. These improvements are clinically meaningful, especially in the context of a study that did not mandate study site visits, face-to-face clinician consultations, changes to diabetes medications, or the initiation of CGM. It is noteworthy that our finding of a − 0.56% reduction in A1c at 3 months in subjects using the OTVR meter with Fitbit is consistent with prior studies of T2D management interventions that incorporated Fitbits, which have reported reductions of − 0.95% to − 1.8% at 3 months^6,13^.

Furthermore, it is worth highlighting that, at baseline, over 25% of our entire cohort were using an incretin medication, and 20% were using an SGLT-2 inhibitor, with high medication adherence (80% or better) self-reported by 77% of the subjects, and still met our inclusion criterion of an A1c 7.5% or higher (see Digital Supplement 2), illustrating the difficulty PwT2D have in managing diabetes and/or weight, despite their access to these newer, effective, and relatively costly medications. A recent review noted that factors such as sedentary lifestyles, unhealthy eating patterns, obesity, urbanization, and limited access to healthcare are fuelling the prevalence of T2D, which accounts for nearly 90% of all people with diabetes^14^. A retrospective analysis of NHANES data from 2009 to 2020 found only 25% of PwT2D using mealtime insulin, and only 12% of those using basal insulin, achieved the ADA A1c goal of < 7%, demonstrating that challenges remain and further solutions are necessary^15^.

Our data supports the view that engagement with relatively low cost and widely accessible digital health and wellness therapeutics can move the dial on type 2 diabetes management in a positive direction. In fact, our entire cohort independently set up and self-trained on their new BGM and synced their BGM via Bluetooth to the OTR diabetes app, which they had downloaded to their mobile phone. Additionally, subjects downloaded the app needed for their chosen digital therapeutic to their phone and, where necessary, synced this health app directly to the OTR app. This scalable and real-world approach to delivering healthcare demonstrates that therapeutics aimed towards lifestyle interventions are effective at delivering better diabetes management.

Although A1c continues to be the gold standard endpoint in most diabetes trials, it is also becoming commonplace to evaluate day to day glycemia in terms of readings in range (RIR) and readings in tight range (RITR). We recently published a real-world evidence (RWE) analysis from more than 55,000 people of Medicare age with type 2 diabetes using the OTR app with a Bluetooth connected OneTouch meter, and found that after 6 months, RIR and RITR improved by + 9.3 and + 10.9%pts, respectively^16^. This evidence, derived from our OTR “data lake,” demonstrates the impact of these advanced monitoring technologies, although we acknowledge that a proportion of these subjects could also have been using other health apps.

In keeping with this large RWE analysis, we sought to understand changes in glucose profiles in our ECLIPSE subjects by comparing their first 14 days of data collection to the 14 days before their 3-month timepoint. However, we faced some study-related challenges with this RWE approach. Notably, subjects may have been more likely to increase engagement and check their BG readings more frequently in the days after first starting a new BGM with multiple colour screens and paired with a new diabetes management app that aggregates their data. It is conceivable that the first 14 days of BG data represented a better diabetes management scenario than a pre-study glucose true baseline would have, which may have obscured additional improvements when we compared to the 14 days before the 3-month timepoint.

Despite this scenario, subjects who chose Noom in combination with OneTouch diabetes management products lowered their mean blood glucose by − 21.8 mg/dL and improved RITR by + 13.7%pts and those using Cecelia Health in combination with OneTouch diabetes management products lowered their mean blood glucose by − 24.5 mg/dL. The Noom and Cecelia Health groups had a relatively larger proportion of people who met our analysis criterion for glycemia changes (of testing at least 5 times per week) compared to subjects in our Fitbit or Welldoc groups, which facilitated detecting these glucose changes. Encouragingly, all four groups witnessed trends towards improvements in key glycemic metrics, including RIR, RITR and hyperglycemic readings, while hypoglycemic readings remained at low levels or were unchanged. These trends toward improvement did not reach statistical significance, which may, in part, be related to the smaller number of subjects remaining after excluding those checking their BG too infrequently to give reliable data for this sub-analysis.

Another aspect to consider is that we did not mandate any increase in, or schedule for, BG check frequency (which remained around 1.5 checks per day). We felt it was more compelling to understand the additional benefit of a new BGM and digital health therapeutic without confounding by a higher BG check frequency than subjects had previously performed. Arguably, if subjects had checked their BG more often, or better still, had performed structured BG monitoring at specific times, then our results may have been even better. A recent meta-analysis in non-insulin-using PwT2D found an additional benefit of − 0.29% in A1c when using structured BG monitoring^17^, and Bergenstal et al.^18^ found that PwT2D who performed structured BG monitoring achieved similar improvements in time in range and A1c to those using CGM.

Ongoing engagement with digital therapeutic interventions is crucial to sustain the positive behavior change driving improved clinical outcomes. We witnessed promising weekly engagement metrics with the Noom and Fitbit interventions. The relationship between app engagement and glycemia will be further explored in future analyses. Unique to the Cecelia Health group were the therapeutic and lifestyle goals agreed upon directly between the Cecelia Health coaches and subjects during their online chat and telephone conversations. We have described the topics covered during conversations and the positive metrics but did not explore the specific personal goals to respect privacy.

In surveys conducted at 3 months, 78–89% of ECLIPSE subjects would recommend their therapeutic intervention to others, with 85% (44/52) of subjects recommending OneTouch devices combined with Noom, 89% (24/27) recommending OneTouch devices with Fitbit, 87% (34/39) recommending OneTouch devices with Cecelia Health and 78% (29/37) recommending OneTouch devices with Welldoc. These findings are consistent with a recent study describing the willingness of people recently diagnosed with T2D to adopt lifestyle or medication changes to manage their diabetes, with 73% willing to engage in healthy eating, 73% in sufficient physical activity and 72% accepting of taking diabetes medications. However, fewer than 50% of those respondents agreed they would adopt all three lifestyle or management options^19^.

Our study does have a number of strengths, such as direct-to-patient recruitment via social media and email rather than recruitment being driven by study coordinators and clinicians. We allowed subjects the freedom to choose their intervention rather than utilizing a forced randomization scheme. The subjects also independently set up and paired their devices to the respective apps. The real-world nature of the recruitment, assignment and technology on-boarding processes closely mirror how consumers and PWDs typically engage with digital therapeutics at home, suggesting broad applicability of these study results.

There are also limitations with our approach. Because we purposefully avoided forced randomization to ensure a more real-world “choice based” approach, a potential for selection bias of subject-driven intervention choices could have resulted in higher engagement and improved outcomes using that chosen intervention than if a subject was forced to use a less desirable (to them) intervention. We feel the ability to mirror these free choices in the real-world outweighed this bias and was a core principle of our design. We aimed for a large cohort by sending study information to thousands of potential subjects, and although over 900 people met screening criteria, 300 of them did not return the mail-in A1c test kits. Furthermore, we did not anticipate the number of exclusions solely due to A1c criteria (the vast majority being below our lower bound A1c threshold of 7.5%). We also found that although subjects engaged with our digital technologies, a sizeable number (26%) did not complete their final A1c measurement at 3 months, reducing our final analysis dataset. This has the potential to introduce bias into the study if, for example, subjects who observed limited improvement in their blood glucose readings disproportionately chose not to complete their A1c at 3 months. While we could not confirm the subjects’ rationales for non-returns, it should be noted that in a US study of PwT2D examining successful completion of at-home A1c kits, the test failure rate was 21 to 26%^20^ due to technical errors, even with live remote assistance from research staff. Therefore, our observation of 26% non-returns is in line with these findings. It is also unclear if the subjects changed their diabetes medications during the study, although our medication adherence surveys did not detect adherence differences between baseline and 3-months. However, the short duration of the study limited the opportunity for medication changes to impact the primary outcome of A1c improvement. A follow-up study would be impactful to allow the subjects to experience the interventions for at least 6 months to confirm outcomes persisted. Furthermore, a comparison of our BGM plus digital therapeutics approach to subjects using CGM would be insightful given that CGM use is increasingly common in PwT2D.

In conclusion, we observed clinically meaningful A1c improvements using the latest Bluetooth connected blood glucose monitoring devices in tandem with digital therapeutics in people with T2D. The scale of the diabetes epidemic necessitates widely accessible, affordable, and engaging interventions that target the very behaviors and knowledge deficits that so often undermine patient progress. Our study provides a practical blueprint for advancing patient care with a choice of therapeutic interventions that can meet the needs of people with type 2 diabetes.

## Electronic supplementary material

Below is the link to the electronic supplementary material.

Supplementary Material 1

Supplementary Material 2

## Data Availability

The datasets used for this study are available from the corresponding author on reasonable request.
