# Supplementary material for: Improved glycemic outcomes in people with type 2 diabetes using smart blood glucose monitoring integrated with popular digital health therapeutics
Source: Sci Rep. 2025 Mar 14;15:8871. doi: 10.1038/s41598-025-93605-1 (PMC11909182; doi:10.1038/s41598-025-93605-1)
Supplement: Supplementary file 1 — Supplementary Material 1 [file 41598_2025_93605_MOESM1_ESM.pdf]

Digital Supplement 1 – ECLIPSE study

A representative selection of mobile phone app (or online web app) screen images from OneTouch and our four digital therapeutic partners are shown below.

Noom app

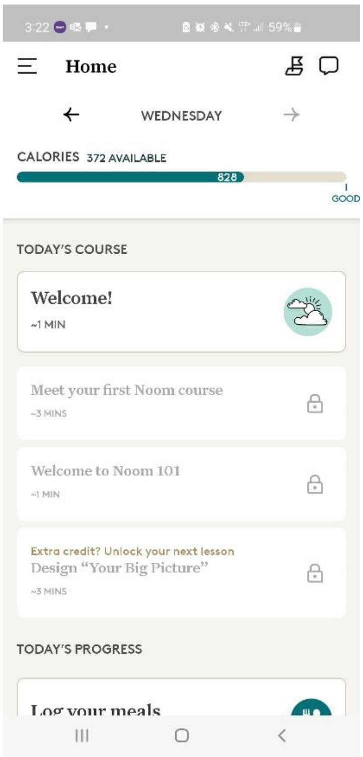

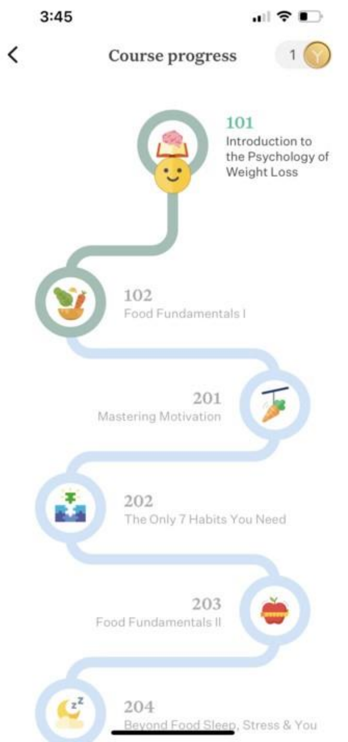

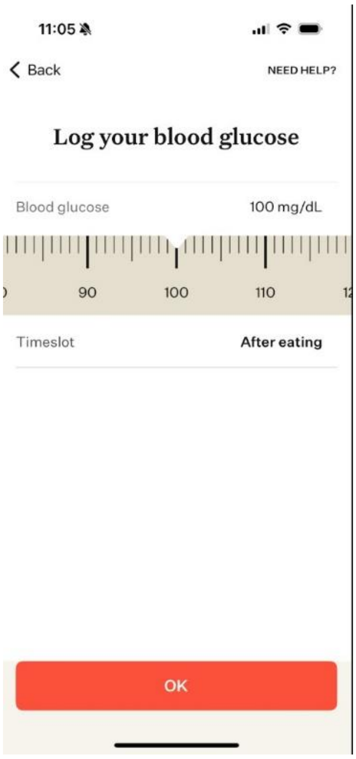

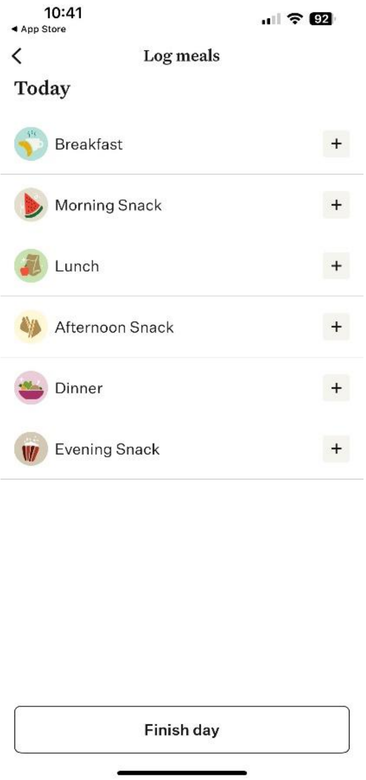

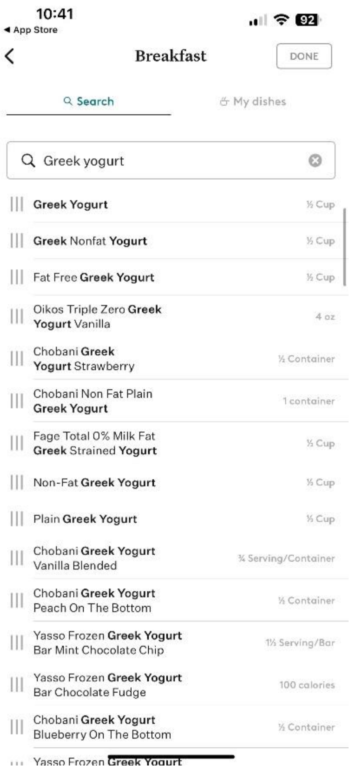

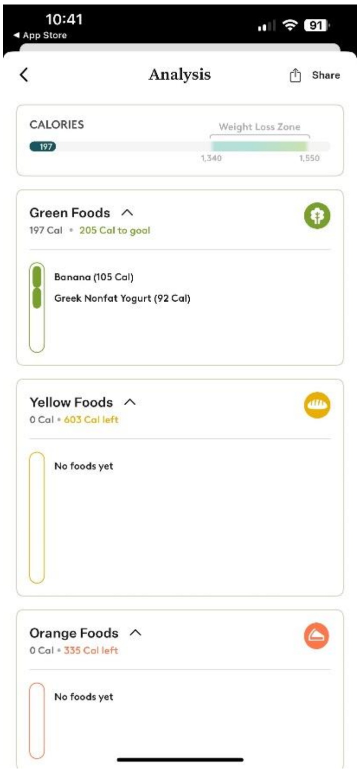

OneTouch® Solutions Fitbit Program

Fitbit premium app

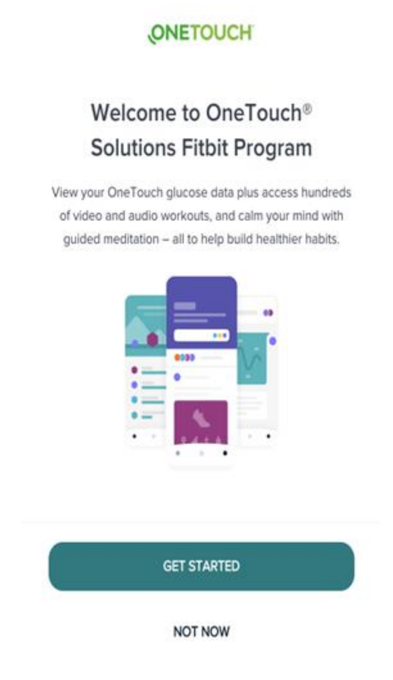

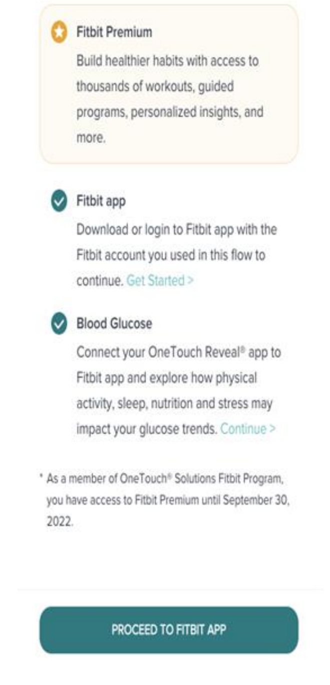

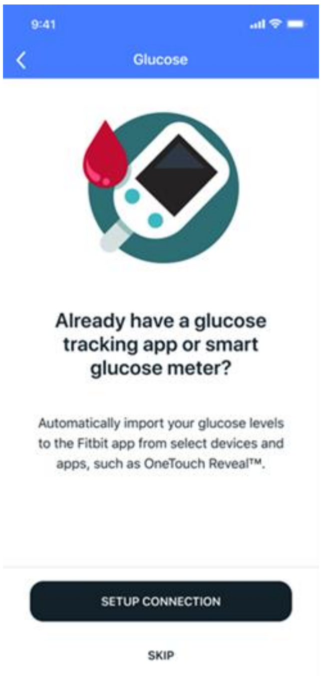

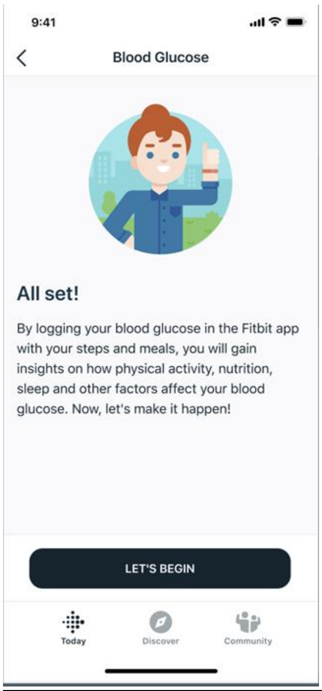

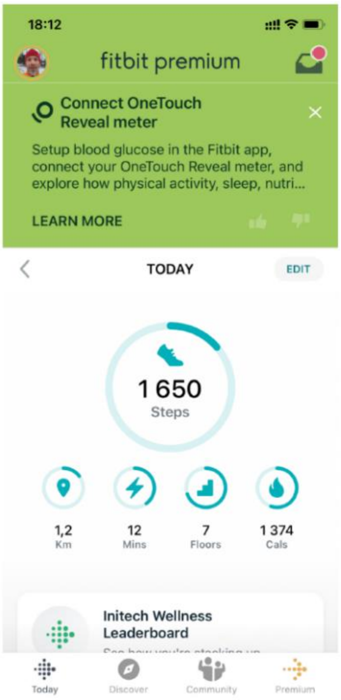

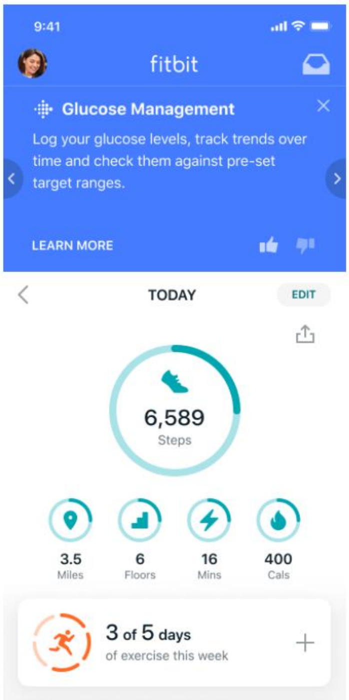

These 3 images show the main Fitbit premium blood glucose tracking screens used by ECLIPSE subjects

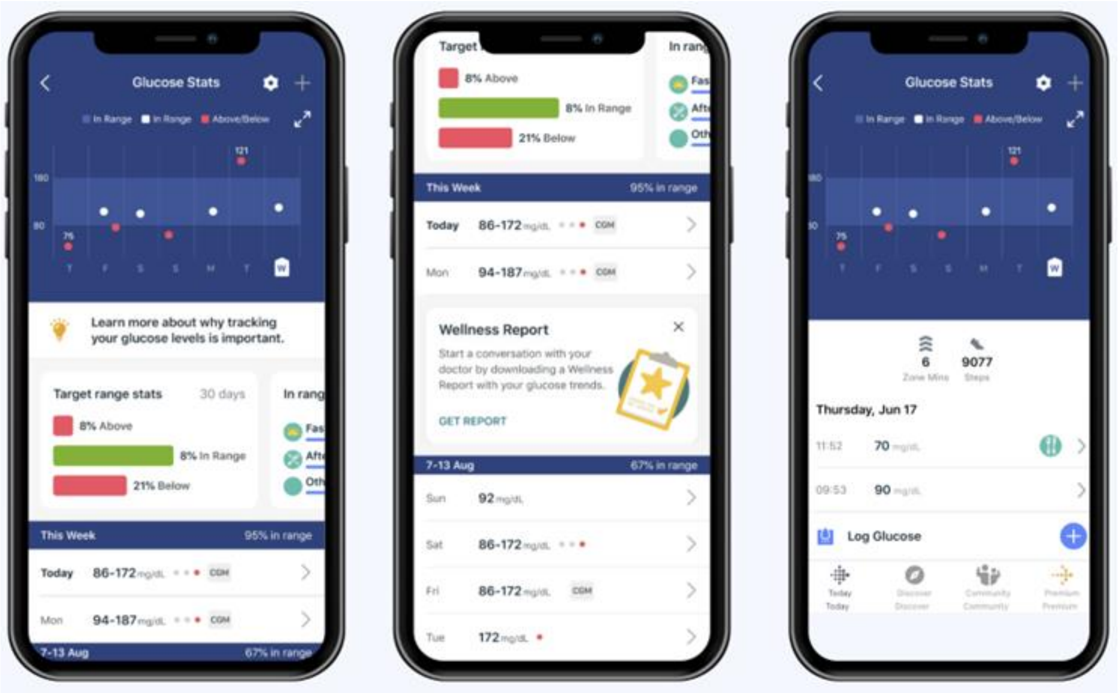

Cecelia Health

Subjects connected to Cecelia coaches via the OneTouch Reveal (OTR) chat function on their mobile phone or using the OTR web portal. Subjects could also talk live to coaches on their phone if a call had been scheduled. Cecelia subjects entered an activation code into OTR app to activate chat with coaches (see images below)

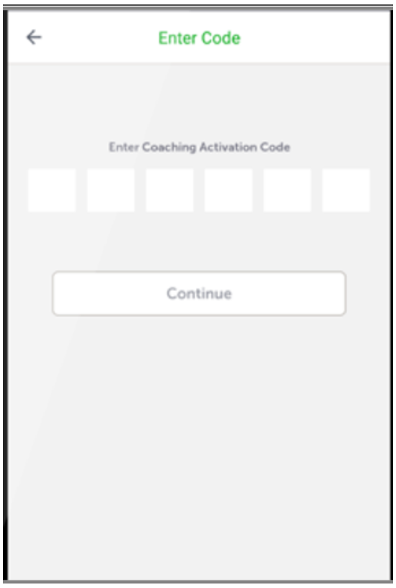

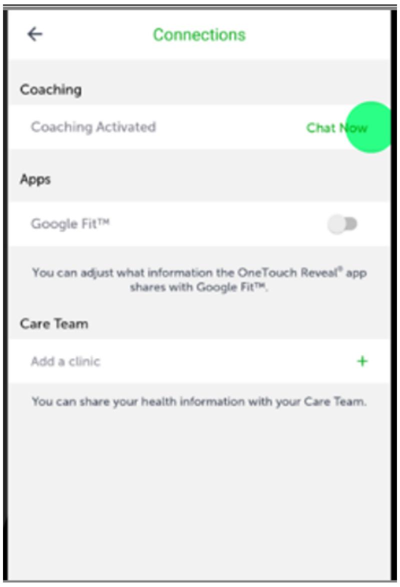

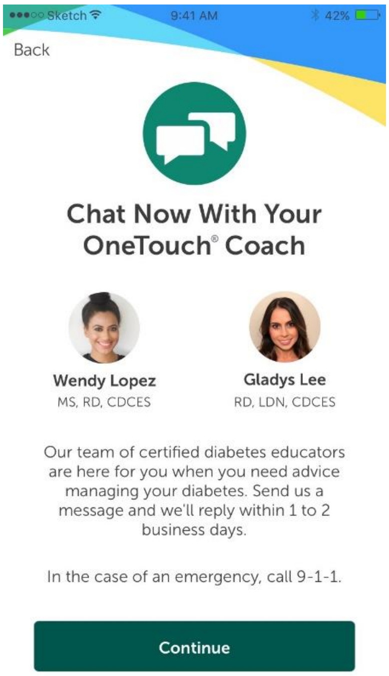

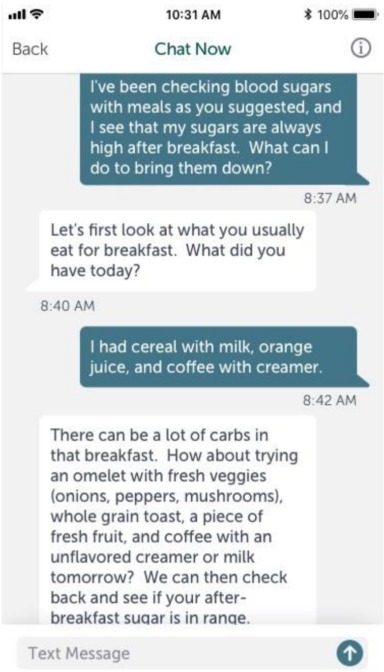

Subjects themselves could also share a summary PDF report with their Cecelia coach (and coaches could also (at will) view this report for subjects who activated coaching)

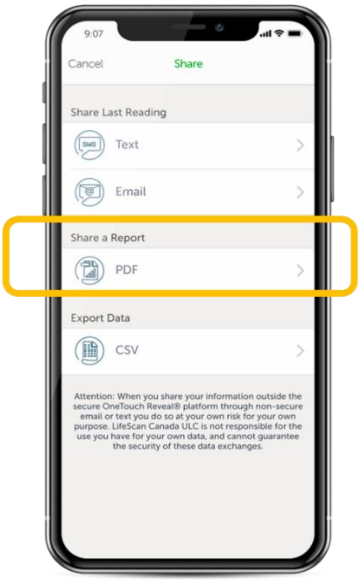

Share Progress

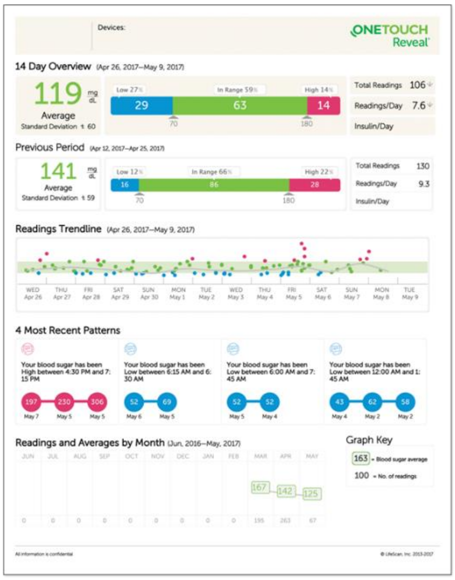

Coaches could also view detailed reports after setting up a clinic account to view information for subjects who had consented to share their information

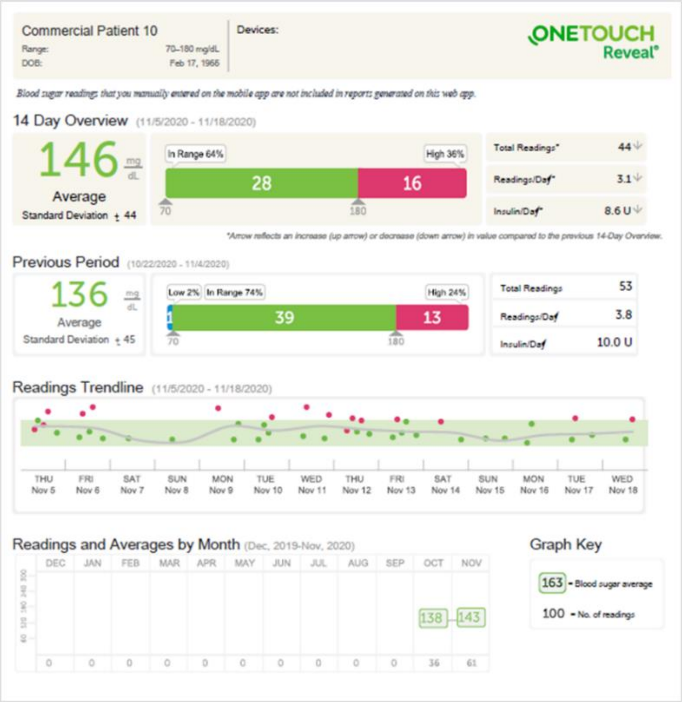

The professional online version of the OneTouch Reveal app allowed Cecelia coaches to track progress of multiple subjects and view more detailed diabetes management reports

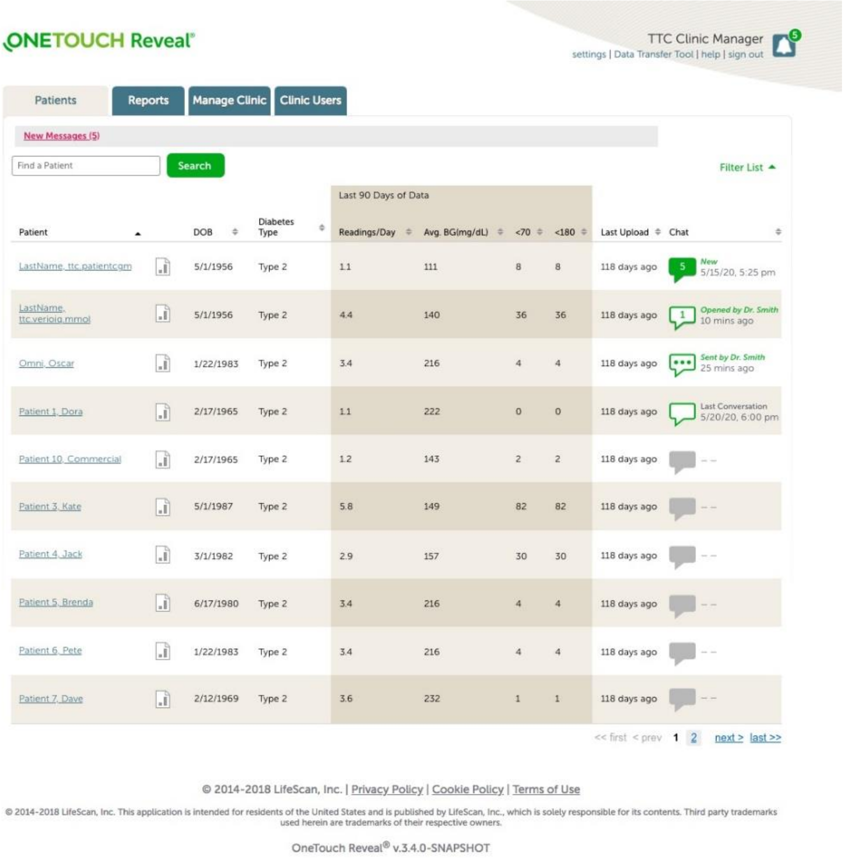

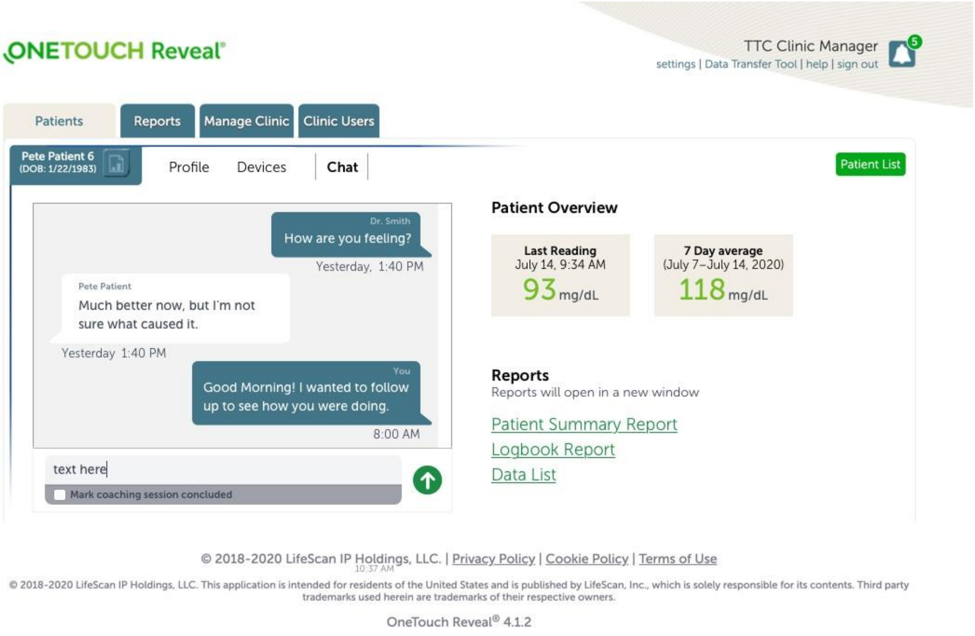
ONETOUCH Reveal®
TTC Clinic Manager
settings | Data Transfer Tool | help | sign out
Patients
Reports
Manage Clinic
Clinic Users
Pete Patient 6
(DOB: 1/22/1983)
Profile
Devices
Chat
Patient List
Dr. Smith
How are you feeling?
Yesterday, 1:40 PM
Pete Patient
Much better now, but I'm not sure what caused it.
Yesterday 1:40 PM
You
Good Morning! I wanted to follow up to see how you were doing.
8:00 AM
text here
Mark coaching session concluded
Patient Overview
Last Reading
July 14, 9:34 AM
93 mg/dL
7 Day average
(July 7–July 14, 2020)
118 mg/dL
Reports
Reports will open in a new window
Patient Summary Report
Logbook Report
Data List
© 2018-2020 LifeScan IP Holdings, LLC. | Privacy Policy | Cookie Policy | Terms of Use
© 2018-2020 LifeScan IP Holdings, LLC. This application is intended for residents of the United States and is published by LifeScan, Inc., which is solely responsible for its contents. Third party trademarks used herein are trademarks of their respective owners.
OneTouch Reveal® 4.1.2

Welldoc app

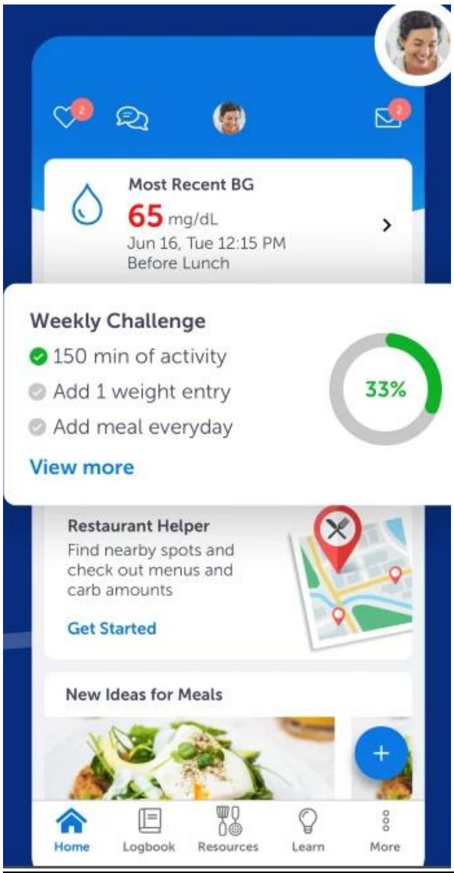
Most Recent BG
65 mg/dL
Jun 16, Tue 12:15 PM
Before Lunch
Weekly Challenge
150 min of activity
Add 1 weight entry
Add meal everyday
33%
View more
Restaurant Helper
Find nearby spots and check out menus and carb amounts
Get Started
New Ideas for Meals
Home
Logbook
Resources
Learn
More

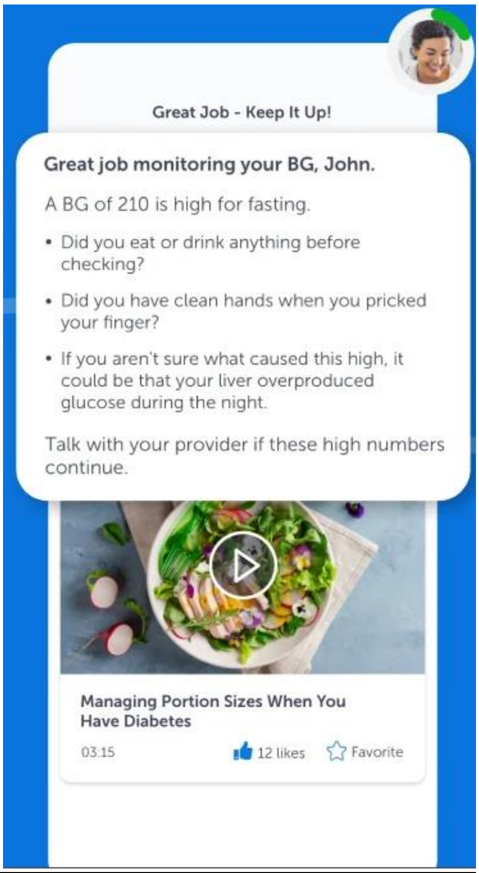
Great Job - Keep It Up!
Great job monitoring your BG, John.
A BG of 210 is high for fasting.
• Did you eat or drink anything before checking?
• Did you have clean hands when you pricked your finger?
• If you aren't sure what caused this high, it could be that your liver overproduced glucose during the night.
Talk with your provider if these high numbers continue.
Managing Portion Sizes When You Have Diabetes
03:15
12 likes
Favorite

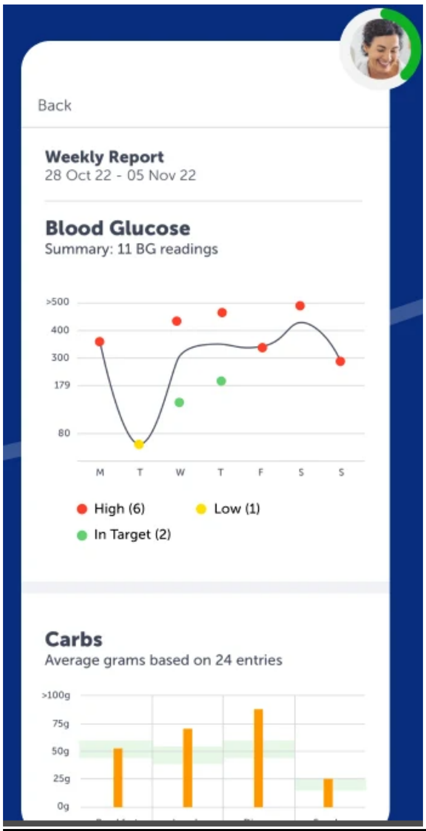
Back
Weekly Report
28 Oct 22 - 05 Nov 22
Blood Glucose
Summary: 11 BG readings
>500
400
300
179
80
M
T
W
T
F
S
S
High (6)
Low (1)
In Target (2)
Carbs
Average grams based on 24 entries
>100g
75g
50g
25g
0g

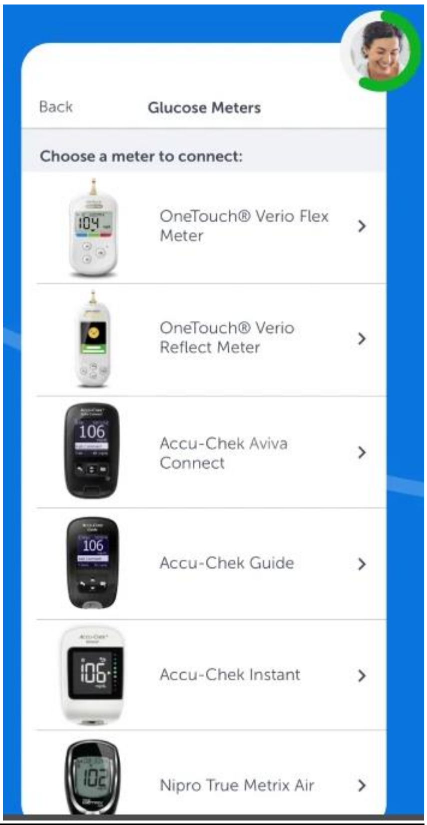
Back
Glucose Meters
Choose a meter to connect:
OneTouch® Verio Flex Meter
OneTouch® Verio Reflect Meter
Accu-Chek Aviva Connect
Accu-Chek Guide
Accu-Chek Instant
Nipro True Metrix Air

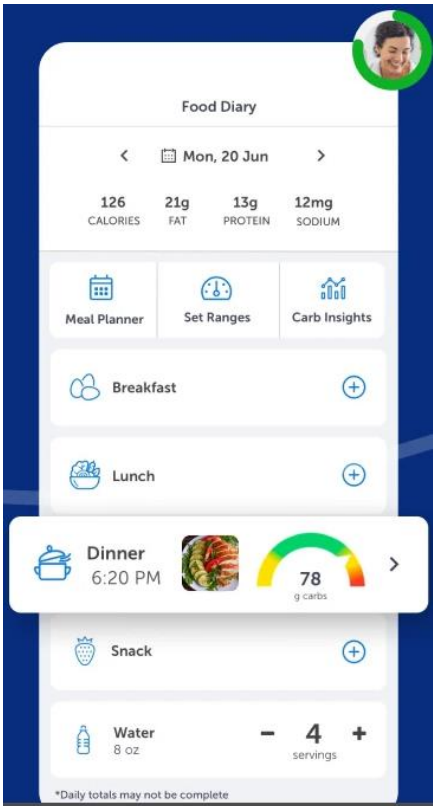
Food Diary
Mon, 20 Jun
126
CALORIES
21g
FAT
13g
PROTEIN
12mg
SODIUM
Meal Planner
Set Ranges
Carb Insights
Breakfast
Lunch
Dinner
6:20 PM
78
g carbs
Snack
Water
8 oz
4
servings
*Daily totals may not be complete
