# Supplementary material for: Improved glycemic outcomes in people with type 2 diabetes using smart blood glucose monitoring integrated with popular digital health therapeutics
Source: Sci Rep. 2025 Mar 14;15:8871. doi: 10.1038/s41598-025-93605-1 (PMC11909182; doi:10.1038/s41598-025-93605-1)
Supplement: Supplementary file 2 — Supplementary Material 2 [file 41598_2025_93605_MOESM2_ESM.pdf]

<u>Digital Supplement 2 – ECLIPSE Study</u>

Additional details on self-reported medications and subject co-morbidities are provided in Table 1 and Table 2

Table 1 – Medications Summary

	Combined (n=191)	Noom (n=68)	Fitbit (n=31)	Cecelia (n=47)	Welldoc (n=45)
Metformin	64% (n=122)	65% (n=44)	61% (n=19)	66% (n=31)	62% (n=28)
Sulfonylurea	32% (n=62)	25% (n=17)	35% (n=11)	43% (n=20)	31% (n=14)
GLP-1 injectable	26% (n=49)	25% (n=17)	29% (n=9)	28% (n=13)	22% (n=10)
SGLT2	20% (n=38)	22% (n=15)	13% (n=4)	21% (n=10)	20% (n=9)
DPP-4	6.3% (n=12)	7.4% (n=5)	6.5% (n=2)	2.1% (n=1)	8.9% (n=4)
TZDs (Glitazones)	4.7% (n=9)	1.5% (n=1)	3.2% (n=1)	2.1% (n=1)	13.0% (n=6)
Meglitinides	1.6% (n=3)	-	-	2.1% (n=1)	4.4% (n=2)
GLP-1 oral	1.6% (n=3)	2.9% (n=2)	-	-	2.2% (n=1)
Other oral meds	13% (n=25)	10% (n=7)	16% (n=5)	19% (n=9)	8.9% (n=4)
Other injectable	3.7% (n=7)	-	6.5% (n=2)	4.3% (n=2)	6.7% (n=3)
Basal insulin (Determir, Glargine, degludec, NPH, other)	40% (n=76)	35% (n=24)	32% (n=10)	30% (n=14)	62% (n=28)
Unsure I take Basal Insulin	3.7% (n=7)	4.4% (n=3)	6.5% (n=2)	4.3% (n=2)	-
Bolus insulin (Aspart, lispro, regular human, other)	15% (n=28)	18% (n=12)	3.2% (n=1)	13% (n=6)	20% (n=9)
Don't take oral meds	9.9% (n=19)	8.8% (n=6)	9.7% (n=3)	11% (n=5)	11% (n=5)
Don't take basal insulin	60% (n=115)	65% (n=44)	61% (n=19)	68% (n=32)	44% (n=20)
Don't take bolus insulin	83% (n=158)	81% (n=55)	94% (n=29)	87% (n=41)	73% (n=33)

Table 2 – Co-morbidities Summary

	Combined (n=191)	Noom (n=68)	Fitbit (n=31)	Cecelia (n=47)	Welldoc (n=45)
Obesity	66% (n=126)	60% (n=41)	61% (n=19)	79% (n=37)	64% (n=29)
Hypertension	63% (n=121)	59% (n=40)	65% (n=20)	62% (n=29)	71% (n=32)
Hyperlipidemia	49% (n=94)	46% (n=31)	58% (n=18)	45% (n=21)	53% (n=24)
Depression	30% (n=58)	32% (n=22)	26% (n=8)	32% (n=15)	29% (n=13)
Asthma	15% (n=30)	15% (n=10)	29% (n=9)	11% (n=5)	8.9% (n=4)
Hypo/hyperthyroid	11.5% (n=22)	6.8% (n=6)	16.2% (n=5)	19.3% (n=9)	4.4% (n=2)
Cardiovascular disease	8.9% (n=17)	5.9% (n=4)	13% (n=4)	15% (n=7)	4.4% (n=2)
Cancer	6.8% (n=13)	7.4% (n=5)	6.5% (n=2)	6.4% (n=3)	6.7% (n=3)
Atrial fibrillation	2.6% (n=5)	4.4% (n=3)	3.2% (n=1)	-	2.2% (n=1)
Heart failure	2.6% (n=5)	1.5% (n=1)	3.2% (n=1)	4.3% (n=2)	2.2% (n=1)
Rheumatoid arthritis	2.6% (n=5)	-	-	6.4% (n=3)	4.4% (n=2)
Chronic kidney disease	2.1% (n=4)	2.9% (n=2)	3.2% (n=1)	-	2.2% (n=1)
COPD	1.6% (n=3)	-	6.5% (n=2)	2.1% (n=1)	-
None of the above	6.3% (n=12)	5.9% (n=4)	6.5% (n=2)	8.5% (n=4)	4.4% (n=2)
